# Arresting Early Childhood Caries with Silver Diamine Fluoride Gel Among Preschool Children: Protocol for a Randomised Clinical Trial

**DOI:** 10.3390/dj12120419

**Published:** 2024-12-22

**Authors:** Anthony Yihong Cheng, Jieyi Chen, Faith Miaomiao Zheng, Duangporn Duangthip, Chun Hung Chu

**Affiliations:** 1 Faculty of Dentistry, The University of Hong Kong, Hong Kong SAR 999077, China; ayhcheng@connect.hku.hk (A.Y.C.); zhengmm@connect.hku.hk (F.M.Z.); 2 Guanghua School of Stomatology, Hospital of Stomatology, Sun Yat-sen University, Guangzhou 510000, China; 3 College of Dentistry, The Ohio State University, Columbus, OH 43210, USA; duangthip.2@osu.edu

**Keywords:** silver, fluoride, gel, caries, children, prevention, clinical trial

## Abstract

**Background**: The World Health Organisation (WHO) included silver diamine fluoride (SDF) in the WHO Model List of Essential Medicines for the management of early childhood caries. SDF is typically available as a 38% aqueous solution, which is watery to apply. A 38% SDF gel has recently been developed, but its caries-arrest effectiveness remains unsubstantiated. The objective of this study is to determine whether the efficacy of a 38% SDF gel is non-inferior to a 38% SDF solution in arresting early childhood caries. **Methods**: This is a 30-month, randomised, active-controlled, parallel-group non-inferiority pragmatic clinical trial with two arms. The hypothesis is that the 38% SDF gel is not worse than the 38% SDF solution by a non-inferiority margin of 10% caries-arrest rate when applied semi-annually to preschool children. This trial will recruit 630 3-year-old kindergarten children through block randomisation to receive either an application of SDF gel or SDF solution on cavitated carious lesions in their primary teeth every 6 months. The primary outcome is the proportion of soft (active) carious tooth surfaces that turn hard (arrested) at the 30-month follow-up. The same calibrated dentist will conduct 6-monthly dental examinations in the kindergartens to assess the status of carious lesions over 30 months. The examiner, the children, and parents will be blinded to treatments. The parents will be surveyed on their child’s oral health-related behaviours and socioeconomic background to allow adjustment for effect modification. **Results:** If the anticipated results are obtained, clinicians can use the 38% SDF gel as an alternative of the 38% SDF solution in arresting early childhood caries. **Conclusions:** As SDF gel is cost-effective, non-invasive, and non-aerosol-generating, it can be widely recommended for caries control. **Trial registration:** ClinicalTrials.gov NCT06241261. Registered on 7 February 2024.

## 1. Introduction

### 1.1. Early Childhood Caries

Early childhood caries (ECC) is the single most common chronic childhood disease worldwide, and its high prevalence is a public health concern. The Global Burden of Disease study (2015) found that untreated ECC affected 573 million children worldwide [1]. In the United States, nearly half of all children had ECC before entering kindergarten [2], and the ECC prevalence could be as high as 98% in some parts of Canada [3]. Due to the increasing global prevalence of ECC among children aged 2–5 years, the FDI World Dental Federation has made this age group a priority [4].

ECC has adverse consequences for children’s health. Untreated dental carious lesions progress and cause local infection which can spread systemically. However, many morbid events related to ECC likely go unreported. Children with ECC are more likely to experience severe caries in adulthood and develop persistent dental fear [5,6]. In both developed and developing countries, ECC is often the main reason for school absences and tooth pain, which has an impact on children’s school performance [7,8]. ECC management is critical to the well-being of children. Traditional restorative methods for preventing and treating ECC are not widely available or affordable in Hong Kong and many countries or regions. Moreover, alternative treatments sometimes need to be administered in hospitals under general anaesthesia, which is extraordinarily costly and may exposes children to unnecessary risks [9]. The FDI Oral Health Atlas reported that millions of children in different parts of the world suffer from untreated ECC, which affects their health, well-being, and development [10]. Considering the significant impact of untreated ECC, it is urgent to develop effective and accessible treatment options.

### 1.2. Silver Diamine Fluoride

Silver diamine fluoride (SDF) is effective in managing ECC. SDF is an effective, efficient and equitable agent that can be adopted to control dental caries [11]. It is now the standard oral care for dental caries in the United States and some countries [12]. SDF therapy is safe, simple, painless, and inexpensive [13]. Treatments can be provided in kindergartens without complicated procedure or expensive equipment [14]. In 2021, the World Health Organisation (WHO) added SDF to the WHO Model List of Essential Medicines [15]. This list represents the most efficacious, safe, and cost-effective medicines for priority conditions.

SDF is currently available as a non-viscous 38% solution [16]. However, SDF solution is not easy to apply and can be easily diluted or washed away by saliva. Moreover, the aqueous solution can easily spill and stain the child’s lips and face. To solve the problem, a 38% SDF gel has recently been developed and can be applied topically. An in vitro qualitative study reported that the 38% SDF gel could penetrate and occlude dentinal tubules, which is necessary for its clinical effectiveness [17]. The gel can be applied quickly and precisely to the carious lesions with less difficulty. To date, there have been no clinical trials that have tested the effectiveness of SDF gel in arresting dental caries, nor have any studies comparatively analysed the efficacy of SDF solution and gel. In theory, the SDF gel should be comparable to the SDF solution in arresting ECC. A well-designed clinical trial is essential to verify that SDF gel is non-inferior to SDF solution in arresting dental caries. Additionally, parental satisfaction, children’s compliance, and any potential adverse effects of the two SDF therapies have also not been investigated. The findings of this study will provide clinical evidence to dental professionals for the adoption of SDF gel in managing ECC.

### 1.3. Objective

The objective of this study is to determine whether the efficacy of a 38% SDF gel is non-inferior to a 38% SDF solution in arresting early childhood caries.

### 1.4. Hypothesis

The hypothesis is that the 38% SDF gel is not worse than the 38% SDF solution by a non-inferiority margin of 10% caries arrest rate when applied semi-annually among preschool children.

### 1.5. Outcome Measures

The primary outcome is the proportion of soft (active) carious tooth surfaces that turn hard (arrested) at the 30-month follow-up. The secondary outcomes are parental satisfaction, children’s compliance, and adverse effects of the SDF therapy.

## 2. Methods/Design

### 2.1. Trial Design

**Figure 1:**
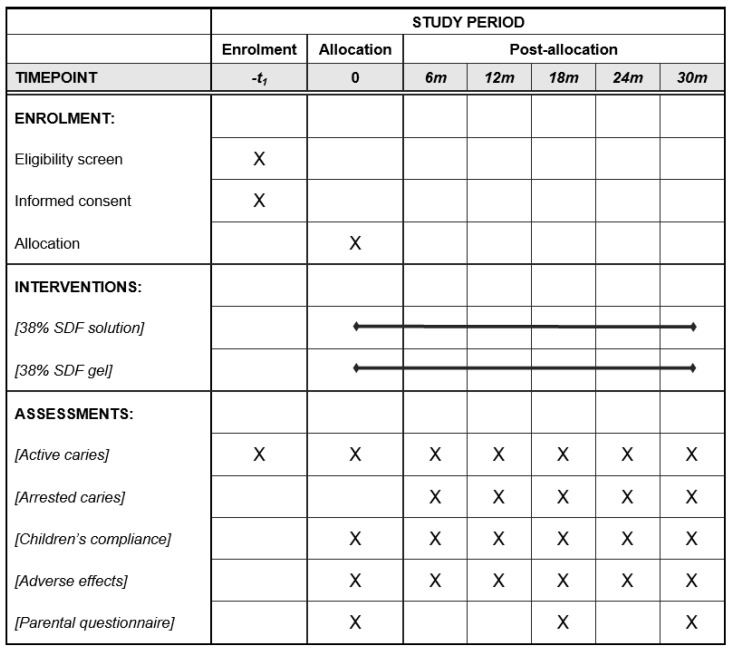
The schedule of enrolment, interventions, and assessments.

This is a 30-month single-centre randomised, active-controlled, parallel-group non-inferiority pragmatic clinical trial with two arms. It follows the Standard Protocol Items: Recommendations for Interventional Trials (SPIRIT) 2013 Statement [18]. Figure 1 shows the trial’s schedule of enrolment, interventions, and assessments. and Supplementary Materials present the SPIRIT 2013 Checklist: Recommended items to address in a clinical trial protocol and related documents.

### 2.2. Ethics Approval and Trial Registration

The trial was approved by the Institutional Review Board of the University of Hong Kong/Hospital Authority Hong Kong West Cluster (UW23-064) on 22 February 2023 and registered in ClinicalTrials.gov (NCT06241261) on 7 February 2024 and Chinese Clinical Trial Registry (ChiCTR2400089113) on 2 September 2024. The trial has been recruiting the first participating child since 1 March 2024 and is expected to complete the recruitment by 31 December 2024.

### 2.3. Setting and Location

This trial will be conducted in kindergartens. The investigators will invite kindergartens which provide outreach dental services. Children aged 3 to 4 years old attending the first year of the recruited kindergarten will be invited to join the trial. An invitation letter explaining the purpose and procedures of the study will be distributed to parents or legal guardians of the children. Kindergarten teachers will collect written informed consent from the parents or guardians prior to the enrolment and pass the consent to the investigators.

### 2.4. Participants

The participants will be children aged 3 to 4 years old who:

(1) are currently attending their first year of kindergarten;

(2) are generally healthy as reported by their parents;

(3) have at least one active carious lesion presented in their primary dentition;

(4) have written informed consents from their parents or legal guardians.

Children will be excluded if they:

(1) are uncooperative impeding the oral examination and/or fluoride therapy;

(2) suffer from major systemic diseases such as porphyria;

(3) are under long-term usage of medications like antiepileptics;

(4) have teeth with dental pulp exposure.

### 2.5. Questionnaire Survey

A parental questionnaire will be administered at the baseline and at the 18-month and 30-month follow-ups [19]. The baseline questionnaire will collect information about children’s oral-health-related behaviour (oral hygiene habits, the use of fluoride agent, parent-assisted tooth-brushing, dental visit behaviour, and snacking habits), and family background information (parental educational level, total family income and family status). The follow-up questionnaires will reassess the children’s oral health-related behaviour and measure parental satisfaction towards their children’s oral health status and appearance by five-point Likert scale. Parents will also be asked to report any potential complications or adverse events experienced by their child after the SDF application, such as tooth pain or gingival irritation.

### 2.6. Clinical Examinations

A trained examiner will perform oral examinations using a WHO periodontal ball-end probe (Otto Leibinger, Mühlheim, Germany) and a front-surface dental mirror with LED illumination (Kudos Crowns Limited, Hong Kong SAR, China) [20]. Tooth status including decayed, missing, and filled tooth surfaces (dmfs) scores, tooth mobility, and discolouration will be recorded. Dental caries will be diagnosed at the cavitation level using visual–tactile assessment. The WHO periodontal ball-end probe will be used to gently explore the centre of suspected carious lesions and carefully avoid any tooth damage. All tooth surfaces will be examined, including the five surfaces of posterior teeth (buccal, lingual, mesial, distal, and occlusal) and the four surfaces of anterior teeth (buccal, lingual, mesial, and distal).

A carious lesion will be classified as active if it is soft during gentle probing. In contrast, if a carious lesion is hard upon probing will be classified as arrested. The assessment of carious lesions is consistent with our previous study [21]. Radiographic imaging will not be performed in this school-based study setting. The examiner will also assess the child’s oral hygiene status using the Visible Plaque Index (VPI) by examining the buccal and lingual surfaces of six index teeth (55, 51, 63, 71, 75, and 83).

Additionally, the presence of visible dental plaque on any existing carious lesions will also be recorded. Intra-examiner agreement will be conducted on 10% of the children who are also the participants of this study at each examination stage to ensure consistency and reliability of the clinical assessments.

### 2.7. Randomisation, Treatment Allocation, and Allocation Concealment

Eligible children will be stratified based on the number of carious tooth surfaces at baseline (1 to 3 surfaces vs. more than 3 surfaces) and randomly allocated to receive either 38% SDF solution or gel. Block randomisation with a block size of 6 will be adopted to assign children to the two treatment groups. A statistician who is not involved in the clinical assessment will generate the randomisation sequence and place the group assignments in opaque sealed envelopes. During the clinical examination, once eligibility is confirmed, a research assistant will assign the randomisation envelope sequentially. This approach ensures effective allocation concealment prior to the administration of the assigned SDF intervention.

### 2.8. Blinding

The examiner, the children, and their parents do not know the intervention allocation. This randomised controlled trial will implement a double-blinding approach to reduce bias. The examiner responsible for conducting the oral examinations and assessing the clinical outcomes will remain blinded to the children’s treatment group assignments throughout the study. The group allocation will be determined by a research assistant not involved in the clinical examinations or outcome assessments. Furthermore, the study children and their parents will not be informed of the assigned treatment group. Unblinding is allowed when a parent requests to know their child’s treatment history. In this case, the child will be regarded as withdrawn from this trial.

### 2.9. Interventions

After the completion of the oral examinations by the blinded examiner at baseline and follow-ups, another dentist will apply the assigned SDF formulation to all carious lesions. According to the recommended clinical procedures, the dentist will first clean away any food debris from the carious lesions and isolate the carious tooth with gauze or cotton roll.

The dentist will dry the carious lesions with a micro-brush and apply the assigned SDF with another micro-brush on the carious lesions for 60 s. Gauze or cotton roll will be used to absorb excessive SDF [22]. The two intervention groups in this study are the following:

Group A: 38% SDF solution (Advantage Arrest 38% SDF solution, Elevate Oral Care, LLC, West Palm Beach, FL, USA) applied semi-annually, and

Group B: 38% SDF gel (Advantage Arrest 38% SDF Gel, Elevate Oral Care, LLC, West Palm Beach, FL, USA) applied semi-annually.

Children in both groups will be instructed to avoid eating or drinking for 30 min after the SDF application. The SDF therapies will be administered every 6 months for a total duration of 30 months. The parents will be informed to report any potential adverse effects, including tooth pain, allergic reactions, gingival irritation, or other issues related to the SDF treatment within 48 h. Referrals for restorative dental treatment will be provided to the children if deemed necessary. Additionally, both teachers and parents will receive information indicating that successfully arrested carious lesions may appear hardened and turn black following the SDF application.

### 2.10. Assessment of the Children’s Compliance

A trained research assistant will assess the children’s compliance during SDF therapy (solution or gel) using Venham’s behaviour-rating scale. This validated instrument provides six scores to evaluate a child’s reactions and behaviours during dental care [23]. Table 1 shows the codes of Venham’s behaviour-rating scale.

### 2.11. Assessment of Harms

An independent, trained health practitioner instead of the examiner will examine each child one day after the SDF therapy. The health practitioner will record the presence of any post-treatment complications, such as pain in the treated tooth, allergic reactions, gingival irritation, or soft tissue staining around the treated tooth. All harms as an unexpected or anticipated event will be reported to the principal investigator. An assigned dentist will provide emergency consultation and dental care for adverse events. An adverse event refers to an untoward occurrence during the study period, which may or may not be causally related to the SDF therapy or other aspects of trial participation. Any severe adverse events involving hospitalisation or serious injury will be reported to the Institutional Review Board within 7 days. In our previous trials, no children were dismissed or excluded because of adverse effects [24].

### 2.12. Follow-Up Evaluations

The follow-up oral examinations will be conducted semi-annually in the kindergarteners for 30 months by the same examiner using the same equipment, standardised procedure, and diagnostic criteria as used in the baseline examination. The examiner will assess the children’s dmfs index, VPI, the status of carious surfaces (classified as active or arrested through gentle probing), and visible dental plaque at carious lesions. New carious lesions will be recorded and treated with the assigned intervention. An independent, trained health practitioner will examine the children for the presence of potential adverse effects related to the SDF treatments one day after the SDF therapy. Children’s compliance with the SDF therapy will be assessed using Venham’s behaviour-rating scale. Any sign of tooth non-vitality, such as tooth hypermobility or dental abscesses, and reasons for premature tooth loss will be documented.

### 2.13. Sample Size and Power Calculation

In our previous clinical trial, the proportion of active carious surfaces that became arrested after the biannual application of 38% SDF solution over 24 months was 76.5% [25]. We set the non-inferiority margin at 7.65% (which corresponds to 10% of the caries arrest rate observed with the SDF solution). This non-inferiority margin represents a difference between the intervention products that we consider to be clinically insignificant. The estimated sample size is based on the lower limit of the two-sided 95% confidence interval for the difference being set above the non-inferiority margin (−7.65%) and the statistical power of the study being set at 90% (β = 0.10) by the Sealed Envelope Power (sample size) calculators [26].

This non-inferiority trial requires 1292 active-caries tooth surfaces at the 30-month follow-up assessment. We have accounted for the fact that each child may present with multiple carious tooth surfaces, with an estimated mean of 4.75 active-carious surfaces per child and an intraclass correlation (ICC) of 0.227, based on our previous study findings [25]. Furthermore, we have estimated a 30-month dropout rate of 20% and an ECC prevalence of 38% among 3-year-old children, according to the available epidemiological data [27]. Therefore, we will screen approximately 3000 3-year-old children to recruit at least 630 children with ECC, with 315 children assigned to each of the two SDF intervention groups.

### 2.14. Data Management

The collected clinical data will be entered into a Microsoft Excel 2019 file (Microsoft Corp., Redmond, WA, USA) independently by two assistants and cross-checked to minimise errors. They will scrutinise any deviations which can be resolved by consensus. Once the data are entered and verified, the statistician will lock the database. The principal investigator will keep the paper forms in locked cabinets and protect all files with passwords to maintain data security.

### 2.15. Statistical Analysis

Data analysis will be conducted using SPSS 24.0 software (SPSS Inc., Chicago, IL, USA). Intra-examiner agreement for caries diagnosis will be evaluated using Cohen’s Kappa statistics. Non-inferiority of the SDF gel group compared with the SDF solution group (control, caries arresting rate 76.5%) will be accepted if the lower limits of the 95% two-sided CI of the difference in the caries-arresting rate is greater than the non-inferiority margin (7.65%). This test for non-inferiority will only be performed for the primary endpoint, which is the proportion of the soft (active) carious tooth lesion surfaces that turn hard (arrested) at the 30-month follow-up. An intention-to-treat approach is planned to utilise all available data from all originally recruited children.

At subject level, chi-square tests will be used to compare proportions of arrested carious surfaces between groups during the follow-ups. These statistical tests will also be applied to analyse the incidence of new carious surfaces and dropout rates between groups during the follow-ups. Independent sample t-tests will be employed to investigate the differences in the mean values of continuous outcome measures, including the number of newly developed carious surfaces, dmfs scores, and non-vital/hypermobile teeth. Descriptive analyses will be used to report the proportions of adverse effects, as well as the levels of child and parental acceptance for each intervention. The McNemar test will be used to compare the changes in oral-health-related behaviours and children’s cooperation between different treatment groups.

A multilevel logistic regression model will be employed to account for the clustering effect of multiple carious lesions within individuals. This multilevel model will assess the effectiveness of 38% SDF gel versus 38% SDF solution in arresting active caries at 6, 12, 18, 24 and 30 months, adjusting relevant covariates, such as social-demographic background, oral health-related behaviours, baseline oral characteristics and daily fluoride exposure. By employing this multilevel analytical framework, the study will provide robust evidence on the efficacy of the 38% SDF gel in managing ECC among preschool children. All hypothesis tests will be two-tailed with an α = 0.05 significance level, and they will be conducted according to the pre-specified analyses, adhering to the intention-to-treat principles. Sensitivity analyses will be performed to address any missing data.

## 3. Discussion

This is a randomised, double-blind, active-controlled, parallel-group clinical trial that aims to investigate the comparative efficacy of semi-annual application of 38% SDF gel and 38% SDF solution for caries management in preschool children. Our objective is to compare the two SDF formulations in their caries-arresting effects over a 30-month period. Additionally, we will assess any adverse effects, parental satisfaction, and children’s compliance. The results of the trial will provide clinical evidence for clinicians to use 38% SDF gel to arrest ECC, especially for children who have difficulty in cooperation during the traditional dental treatment.

While SDF has been widely recognised as a cost-effective, simple, and non-invasive method in arresting carious lesions and a valuable tool in managing ECC, the traditional SDF aqueous solution has some limitations. The liquidity of solution may make the application on the carious lesions precisely challenging, increasing the risk of unintended soft tissue exposure and staining [28]. Additionally, the solution’s unpleasant metallic taste often stimulates salivary secretion, which may dilute or wash away the applied agent before it takes effect. This may diminish the overall caries prevention benefits and may negatively influence children’s experience and acceptance [29]. The 38% SDF gel formulation was therefore developed to address these drawbacks. The viscous, adhesive gel consistency is designed to better maintain the SDF at the carious lesions, reducing the risk of migration to adjacent areas and the potential for adverse effects. The gel formulation is also expected to be more resistant to rapid salivary dilution or mechanical expulsion, potentially improving overall caries arrest efficacy and children’s experience [30]. Notably, the SDF gel does not stain sound dentin or enamel, preserving the aesthetic appearance, though it can stain carious lesions, allowing for better monitoring of dental caries progression or regression [31]. Both the SDF gel and solution feature a blue tint, which enhances user-friendliness during the application process. Additionally, the SDF gel’s odourless nature and mitigation of the metallic taste make it more acceptable to preschool children, further enhancing its practicality in the clinical setting [32].

However, direct comparative evidence on the caries-arresting capacity and clinical efficacy of SDF gel versus the traditional SDF solution is limited. To address this evidence gap, this randomised controlled trial will employ a non-inferiority design to evaluate whether the SDF gel formulation is at least as effective as the standard SDF solution in arresting dental caries. We selected a 10% non-inferiority margin based on the existing literature and practical considerations in paediatric dentistry [33]. This margin allows us to assess clinical relevance while accommodating the challenges associated with sample size in the community. Given that this clinical trial requires the screening of approximately 3000 preschool children, the chosen margin strikes a balance between ensuring meaningful clinical outcomes and maintaining the feasibility of the trial. Furthermore, SDF gel may potentially offer additional benefits, such as improving application precision, reducing soft tissue staining, and enhancing patients’ acceptability. This study has large sample size which will provide adequate statistical power to detect any non-inferiority of the SDF gel compared to the SDF solution. Demonstrating the non-inferiority of SDF gel could support its clinical use, especially for paediatric patients among whom the precisely application of SDF therapies and extended contact time of the agents are prioritised. SDF gel also retains other advantages of SDF, such as affordability, accessibility, minimal invasiveness, and non-aerosol generation, making it an attractive option for public-health-oriented caries prevention and management strategies.

Overall, this study aims to provide robust clinical evidence on the effectiveness of 38% SDF gel in managing ECC. Confirming its non-inferiority to traditional SDF solution would promote its adoption, especially in public health contexts focused on paediatric oral care. The SDF gel’s affordability, ease of use, and minimal invasiveness offer a promising tool for advancing caries prevention strategies and improving children’s oral health worldwide.

## Figures and Tables

**Table 1:** Venham’s behaviour-rating scale.

Score	Behaviour Rating
0	Total cooperation, best possible working conditions, no crying or physical protest.
1	Mild, soft verbal protest, or (quiet) crying as a signal of discomfort, but does not obstruct progress. Appropriate behaviour for procedure.
2	Protest more prominent. Both crying and hand signals. May move head around making it hard to administer treatment. Protest more distracting and troublesome. In contrast, child still complies with request to cooperate.
3	Protest presents real problem to dentist. Complies with demands reluctantly, requiring extra effort by dentist, body movement.
4	Protest disrupts procedure and requires that all of the dentist’s attention be directed toward the child’s behaviour. Compliance eventually achieved after considerable effort by dentist, but without much actual physical restraint.
5	Uncooperative. General protest, no compliance, or cooperation. Physical restraint is required.

## Data Availability

The results of the dental examination of each participating child will be shared with the parents or legal guardians via oral health reports. The team will share the results of the study with academia via publications and presentations. The datasets generated in this trial will be available from the primary investigator on a legitimate request.

## Supplementary Materials

The following supporting information can be downloaded at https://www.mdpi.com/article/10.3390/dj12120419/s1, S1: SPIRIT 2013 checklist; S2: Parental consent form.
