# Supplementary material for: Arresting Early Childhood Caries with Silver Diamine Fluoride Gel Among Preschool Children: Protocol for a Randomised Clinical Trial
Source: Dent J (Basel). 2024 Dec 22;12(12):419. doi: 10.3390/dj12120419 (PMC11674657; doi:10.3390/dj12120419)
Supplement: Supplementary file 1 [file dentistry-12-00419-s001.zip › Supplementary 2_Parental Consent.pdf]

HKU Dentistry
Faculty of Dentistry · The University of Hong Kong
香港大學牙醫學院

Consent form_EN, Version3, 08 February 2023

1st September 2023

K1 only

Dear Parents of preschool children,

Re: Randomised clinical trial to arrest early childhood caries with a silver diamine fluoride gel

The Faculty of Dentistry of the University of Hong Kong is now conducting a clinical study about caries treatment among preschool children. The aims are is to determine whether the efficacy of a 38% silver diamine fluoride (SDF) gel is non-inferior to a 38% SDF solution for arresting carious lesions in preschool children when applied at half-yearly intervals over a 24-month period. Your child's kindergarten is one of the participating kindergartens, and we will provide participating children a 24-month caries preventive and arresting treatment.

The details of the activity would be as follows:

- Target: K1 students;
- Proposed period: Semester year 2023-2026 including routine oral examination and fluoride treatment at six-month intervals
- Activities: (1) oral health education; (2) parental questionnaire survey; (3) oral examination; (4) SDF gel or SDF solution treatment.

These activities will be carried out in your child's kindergarten. Each child will be randomly allocated to a treatment group of SDF gel or SDF solution treatment. The oral examination and treatment will take 5 minutes. No radiographs will be taken. No lifestyle or dietary restriction is required during the study period. Each participating child will receive a report regarding his/her oral health status. Subsequent semi-annual oral examination will be performed for the participants. Those who need any other dental services such as extraction can be treated by their own dentist at their own cost.

This study will provide fluoride treatment only to the carious teeth of your child. The decayed lesion may become black after the fluoride treatment. The black stain will not fade away until the replacement of milk teeth. However, please be noted that the black stain means the tooth decay has been successfully stopped from progressing. SDF may stain grey if accidentally dropped onto skin, but it will disappear within a week's time.

It is up to you to decide whether or not to let your child to take part. If you decide to take part in the study, please fill in the questionnaire attached as well as authorise us to provide a dental examination and fluoride treatment to your children in school time. You are still free to withdraw at any time and without giving a reason. This will not affect the standard of care you receive in the future.

You have the rights of access to personal data and publicly available study results, if and when needed. Under the laws of Hong Kong (in particular the Personal Data (Privacy) Ordinance, Cap 486), you enjoy or may enjoy rights for the protection of the confidentiality of your personal data, such as those regarding the collection, custody, retention, management, control, use (including analysis or comparison), transfer in or out of Hong Kong, non-disclosure,

erasure and/or in any way dealing with or disposing of any of your personal data in or for this study. For any query, you should consult the Privacy Commissioner for Personal Data or his office (tel no. 2827 2827) as to the proper monitoring or supervision of your personal data protection so that your full awareness and understanding of the significance of compliance with the law governing privacy data is assured.

By consenting to participate in this study, you expressly authorise:

a) the principal investigator and his research team and the ethics committee (Institutional Review Board of the University of Hong Kong / Hospital Authority Hong Kong West Cluster) responsible for overseeing this study to get access to, to use, and to retain your personal data for the purposes and in the manner described in this informed consent process; and
b) the relevant government agencies (e.g. the Hong Kong Department of Health) to get access to your personal data for the purposes of checking and verifying the integrity of study data and assessing compliance with the study protocol and relevant requirements.

It is possible that when taking part in the oral examination, your child may feel discomfort. If your child is too unwilling to be checked, the oral examination will stop immediately. If your child is harmed by taking part in this study, there are no special compensation arrangements. If your child is harmed due to someone’s negligence, then you may have grounds for a legal action. Regardless of this, if you wish to complain about any aspect of the way your child has been approached during the course of this study, the University of Hong Kong may be available to you.

After examination, each of the participants will receive a set of souvenir. We hope that the oral examination and fluoride treatment will help you to understand the oral health status of your child. This study has been reviewed by Institutional Review Board of the University of Hong Kong / Hospital Authority Hong Kong West Cluster. If you have any enquiry, please contact me at 2859 0287 during office hours. Please complete the following consent if you agree to participate in this study. Thank you very much.

This research project will be provided for free by the Faculty of Dentistry, HKU, with no commercial interest. We sincerely ask for your approval to allow your child to participate. You are entitled to refuse to participate in this research project and this will not affect your child’s rights of medical and dental services. You can also withdraw from the research any time. All information and data collected will be kept confidential and use for research purpose only. If you have any questions, you are welcome to reach Prof. C.H. Chu by phone 2859 0287 or through e-mail chchu@hku.hk.

Thank you.

Prof. C.H. Chu, Clinical Professor,
Faculty of Dentistry, The University of Hong Kong

PATIENT/SUBJECT CONSENT FORM

Child name: ______________________

Title of Project: **Randomised clinical trial to arrest early childhood caries with a silver diamine fluoride gel**

Name of Researcher: Prof. Chu Chun Hung

Please tick ✓ in the box

1. I confirm that I have read and understood the information sheet for the above study and have had the opportunity to ask questions. ☐

2. I understand that my participation is voluntary and that I am free to withdraw any time, without giving any reason, without my medical care or legal rights being affected. ☐

3. I understand that sections of any of my medical notes may be looked at by responsible individuals from Faculty of Dentistry, The University of Hong Kong or from Institutional Review Board of the University of Hong Kong / Hospital Authority Hong Kong West Cluster.
   I give permission for these individuals to have access to my records. ☐

4. Regarding this three-year study:

   4.1. I agree to let my child undergo oral examination and fluoride treatment; ☐

   4.2. I agree to complete the questionnaire; ☐

   4.3. I agree to have photos taken of the teeth of my child (not including face). ☐

______________________	______________________	______________________
Name of Parent / Guardian	Date	Signature
Prof. Chun Hung Chu	______________________	______________________
Researcher	Date	Signature
